# Endoscopic lithotripsy for an impacted biliary stone at the confluence of the cystic duct and common bile duct using a novel drill dilator

**DOI:** 10.1055/a-2281-9683

**Published:** 2024-04-03

**Authors:** Yuichi Hirata, Yuichiro Aoyama, Ryosuke Mizukami, Mayumi Doi, Atsunori Maeda, Daisuke Orita, Yoshihiro Okabe

**Affiliations:** 1 469536 Department of Gastroenterology, Kakogawa Central City Hospital, Kakogawa, Japan

Endoscopic retrograde cholangiopancreatography (ERCP) is widely performed for patients with common bile duct (CBD) stones. Many cases are successfully managed with endoscopic sphincterotomy and stone removal using balloon or basket catheters. However, for difficult or complex stones, ERCP with conventional techniques may fail to achieve biliary clearance in 10–15% of cases 1 . Peroral cholangioscopy-guided electrohydraulic lithotripsy (POCS-EHL) is effective in these cases; however, it is expensive and time-consuming. Herein, we report a case of endoscopic lithotripsy for an impacted stone at the confluence of the cystic duct (CD) and CBD using a novel drill dilator.

A 77-year-old woman with a high fever and abdominal pain was admitted to our institution. Blood tests showed obstructive jaundice, and computed tomography revealed a common bile duct stone ( Fig. 1 ). ERCP revealed the stone was present at the confluence of the CD and CBD ( Fig. 2 **a** ). Although stone removal using a mechanical lithotriptor (LithoCrush V; Olympus Medical Systems, Tokyo, Japan) was attempted, it was difficult to catch and crush the stone because of the narrow working space ( Fig. 2 **b** ). Although POCS-EHL is an alternative procedure, it is not regularly performed in our institution. Therefore, endoscopic lithotripsy was performed using a novel drill dilator (Tornus ES; ASAHI INTECC, Aichi, Japan and Olympus Medical Systems, Tokyo, Japan) for the confluence stone. With a clockwise rotation, the drill dilator could be passed through, while chipping away at the stone, without requiring a strong pushing force. The stone was completely removed using conventional basket and balloon catheters during a single ERCP session ( Fig. 3 , Video 1 ). It is thought that the twisting force of the drill dilator was easily transmitted to the impacted stone, which has poor mobility, leading to lithotripsy.

**Fig. 1:**
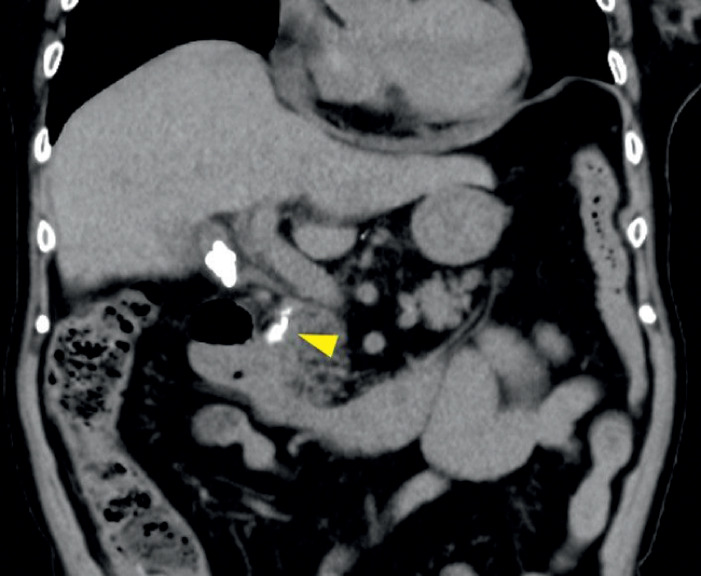
Computed tomography (CT) images. CT revealed a common bile duct stone.

**Fig. 2:**
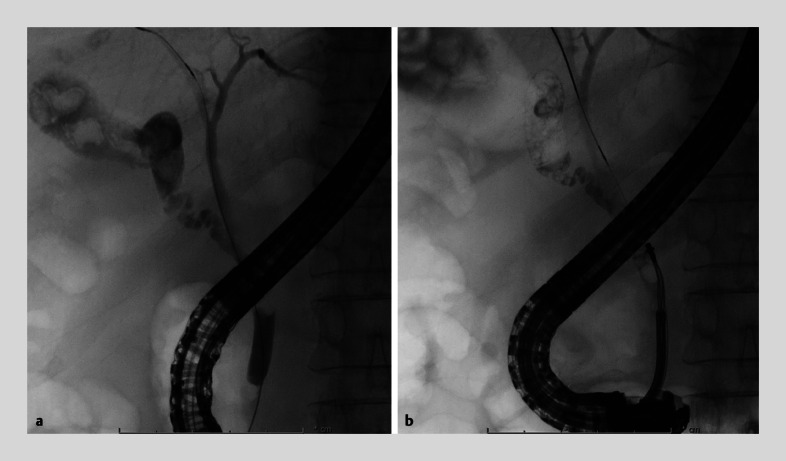
Fluoroscopic images. **a** Endoscopic retrograde cholangiopancreatography (ERCP) revealed the stone was present at the confluence of the cystic duct (CD) and common bile duct (CBD). **b** It was difficult to catch and crush the stone using a mechanical lithotriptor because of the narrow working space.

**Fig. 3:**
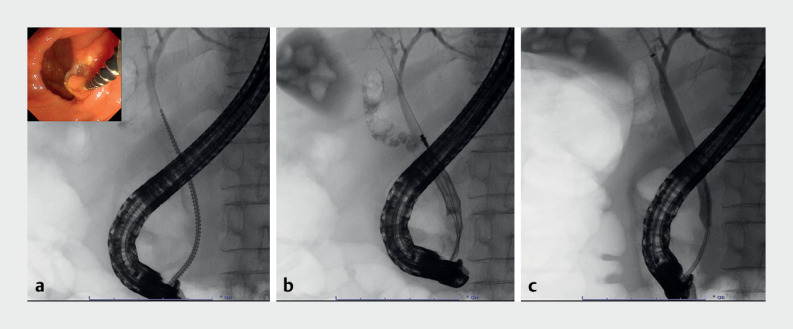
Fluoroscopic images. **a** Endoscopic lithotripsy using a novel drill dilator. **b** Stone removal using a basket catheter. **c** Biliary clearance using a balloon catheter.

Endoscopic lithotripsy for an impacted biliary stone at the confluence of the cystic duct and common bile duct using a novel drill dilator.Video 1

Endoscopic lithotripsy using a novel drill dilator is useful in patients with an impacted biliary stone at the confluence of the CD and CBD.

Endoscopy_UCTN_Code_TTT_1AR_2AH

## Notes

Endoscopy E-Videos https://eref.thieme.de/e-videos
*E-Videos* is an open access online section of the journal *Endoscopy* , reporting on interesting cases and new techniques in gastroenterological endoscopy. All papers include a high-quality video and are published with a Creative Commons CC-BY license. Endoscopy E-Videos qualify for HINARI discounts and waivers and eligibility is automatically checked during the submission process. We grant 100% waivers to articles whose corresponding authors are based in Group A countries and 50% waivers to those who are based in Group B countries as classified by Research4Life (see: https://www.research4life.org/access/eligibility/ ). This section has its own submission website at https://mc.manuscriptcentral.com/e-videos .
